# New Insights into Endothelial Dysfunction in Cardiometabolic Diseases: Potential Mechanisms and Clinical Implications

**DOI:** 10.3390/ijms25052973

**Published:** 2024-03-04

**Authors:** Giovanna Gallo, Carmine Savoia

**Affiliations:** Clinical and Molecular Medicine Department, Faculty of Medicine and Psychology, Sant’Andrea Hospital, Sapienza University of Rome, Via di Grottarossa 1035–1039, 00189 Rome, Italy; giovanna.gallo@uniroma1.it

**Keywords:** endothelium, diabetes, metabolic syndrome, endothelial dysregulation, cardiometabolic diseases

## Abstract

The endothelium is a monocellular layer covering the inner surface of blood vessels. It maintains vascular homeostasis regulating vascular tone and permeability and exerts anti-inflammatory, antioxidant, anti-proliferative, and anti-thrombotic functions. When the endothelium is exposed to detrimental stimuli including hyperglycemia, hyperlipidemia, and neurohormonal imbalance, different biological pathways are activated leading to oxidative stress, endothelial dysfunction, increased secretion of adipokines, cytokines, endothelin-1, and fibroblast growth factor, and reduced nitric oxide production, leading eventually to a loss of integrity. Endothelial dysfunction has emerged as a hallmark of dysmetabolic vascular impairment and contributes to detrimental effects on cardiac metabolism and diastolic dysfunction, and to the development of cardiovascular diseases including heart failure. Different biomarkers of endothelial dysfunction have been proposed to predict cardiovascular diseases in order to identify microvascular and macrovascular damage and the development of atherosclerosis, particularly in metabolic disorders. Endothelial dysfunction also plays an important role in the development of severe COVID-19 and cardiovascular complications in dysmetabolic patients after SARS-CoV-2 infection. In this review, we will discuss the biological mechanisms involved in endothelial dysregulation in the context of cardiometabolic diseases as well as the available and promising biomarkers of endothelial dysfunction in clinical practice.

## 1. Introduction

The endothelium is a monocellular layer covering the inner surface of blood vessels which maintains vascular homeostasis through different protective properties. These include the regulation of vascular tone and permeability and of anti-inflammatory, antioxidant, anti-proliferative and anti-thrombotic functions. The endothelium induces the release of autocrine, paracrine, and endocrine substances [1], including the synthesis of nitric oxide (NO), C-type natriuretic peptide (CNP), prostacyclin, and endothelium-derived hyperpolarizing factor, which contribute to the inhibition of smooth muscle cells proliferation and migration, the inhibition of platelets’ adhesion and aggregation, and the modulation of biological processes leading to thrombogenesis [2,3].

A bidirectional relationship exists between endothelial disfunction and cardiometabolic diseases. All the components of metabolic disorders including abdominal obesity and impaired glycemic control, and hypertension may be associated with endothelial dysfunction [4]. On the other hand, structural and functional alterations of the endothelium contribute to the development of cardiometabolic disease, and endothelial dysfunction contributes to the development of atherosclerosis and may worsen the cardiometabolic disease. In diabetic patients in particular, the glycation of endothelial cells plays a role in the activation of pathophysiological mechanisms leading to inflammation, reactive oxygen species (ROS) production, and impaired synthesis of NO which contribute to atherosclerosis progression, coronary artery disease, carotid artery disease, peripheral artery disease, and ischemic cerebral events [5].

In this review, we will discuss the biological mechanisms involved in endothelial dysregulation in the context of cardiometabolic diseases. Moreover, we will describe the available and promising markers of endothelial dysfunction, which might represent an innovative tool to predict the risk of cardiovascular events, and the therapeutic strategies with potential beneficial effects on endothelial function.

## 2. Cardiometabolic Alterations and Endothelial Dysfunction

Cardiometabolic diseases involves alterations in glucose and lipid homeostasis due to insulin resistance. These metabolic abnormalities may contribute to endothelial dysfunction through different mechanisms. Endothelium serves as a gatekeeper for tissue homeostasis, being involved in the regulation of nutrients and metabolites transport. Moreover, it interacts with circulating cells and hormones, contributing to the maintenance of vascular tone [1,6]. Endothelial dysfunction is a consequence of increased oxidative stress, reduced NO production, increased secretion of adipokines, endothelin-1, and fibroblast growth factor 2 (FGF-2), which stimulate inflammatory pathways, intimal growth, angiogenesis, and proliferation of smooth muscle cells [7,8].

**Figure 1:**
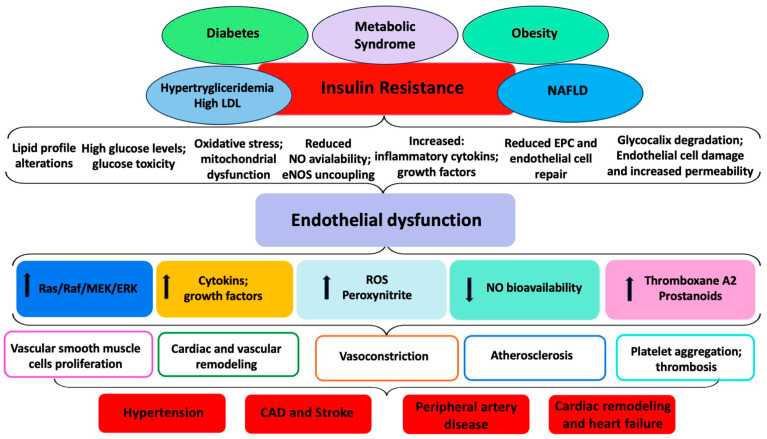
Development of endothelial dysfunction in cardiometabolic diseases: pathophysiological mechanisms. Figure legend: CAD, coronary artery disease; eNOS, endothelial nitric oxide synthase; EPC, endothelial progenitor cells; LDL, low-density lipoprotein; NAFLD, non-alcoholic fatty liver disease; NO, nitric oxide; Ras (Rat Sarcoma Virus) protein; Raf (Rapid Accelerated Fibrosarcoma) protein; MEK, Mitogen-activated protein kinase kinase; ERK, Extracellular-signal-regulated kinase; ROS, reactive oxygen species; ↑ increase; ↓ decrease.

Figure 1 summarizes the pathophysiological mechanisms involved in the development of endothelial dysfunction in cardiometabolic diseases.

### 2.1. Metabolic Syndrome, Insulin Resistance, and Endothelial Dysfunction

Insulin resistance is a key factor in the development of metabolic alterations and endothelial dysfunction. In adults, hyperglycemia is a consequence of peripheral insulin resistance which consists of impaired insulin action on peripheral tissues that results in hyperinsulinemia to attempt to maintain normal glucose levels [9]. Insulin resistance plays a pivotal role in the development of metabolic syndrome, which is defined as a cluster of metabolic abnormalities including hypertension, central obesity, and atherogenic dyslipidemia [10]. In the context of metabolic syndrome, insulin resistance may be associated with an increased release of circulating free fatty acids (FFA) from an expanded adipose tissue mass [11], which further contributes to the metabolic alterations. FFA inhibit insulin-mediated glucose uptake in muscles, resulting in elevated levels of circulating glucose and thus in increased pancreatic insulin secretion. In the liver, FFA increase the production of glucose, triglycerides, and very low-density lipoproteins (VLDL) and reduce the glucose transformation into glycogen [12]. Since insulin exerts antilipolytic actions, insulin resistance is associated with an increased lipolysis of stored triacylglycerol molecules in adipose tissue and an enhanced production of FFA in a detrimental vicious circle [13]. Hypertriglyceridemia contributes to the reduction in the cholesteryl ester content in the lipoprotein core and consequently to the reduction in the protective high-density lipoproteins (HDL) [14]. Moreover, the relative depletion of unesterified and esterified cholesterol and phospholipids contributes to the formation of low-density lipoproteins (LDL) which are small and dense with increased proatherogenic properties [14]. Insulin resistance is also associated with increased levels of apo B and C-III, uric acid, prothrombotic factors such as fibrinogen and plasminogen activator inhibitor 1, homocysteine, heparin-binding epidermal growth factor (EGF)-like growth factor, and pro-inflammatory cytokines, which contribute to endothelial dysfunction and vascular remodeling [14].

Hyperglycemia may induce more serious damages in endothelial cells than in other cell types. In healthy conditions, glucose enters the cell mainly through the GLUT-1 transporter which promotes ATP generation primarily via the glycolytic pathway. GLUT-1 activity is regulated by extracellular glucose concentrations [15,16]. In diabetic patients GLUT-1 activity is enhanced and contributes to the increased glycolytic flux which causes a rise in intracellular glucose concentrations and in advanced glycation end products (AGEs) [15]. This overproduction of AGEs is associated with an increased endothelial cell permeability, inhibition of endothelial NO synthase (eNOS) activity, and enhanced detrimental alterations of DNA and proteins leading to cell damage [15]. Hyperglycemia rapidly activates cellular proliferation in endothelial cells through the stimulation of different proinflammatory and growth factors including the hepatocyte growth factor (HGF), vascular endothelial growth factor (VEGF) family ligand–receptor, Erb-B2 Receptor Tyrosine Kinase 4 (ErbB4), insulin-like growth factor (IGF-1), bone morphogenetic protein (BMP), nuclear factor of activated T-cells (NFAT), signal transducer and activator of transcription 3 (STAT3), nuclear factor-kappaB (NF-κB), p70S6K and hypoxia-inducible factor-1alpha (HIF-1α) signaling pathways [17]. Moreover, hyperglycemia increases the expression of membrane cofactor protein-1 (MCP-1) and NLR family pyrin domain containing 3 inflammasome (NLPR3) and promotes mitochondrial oxidative stress and apoptosis.

Hyperglycemia also decreases the expression of protective and anti-inflammatory factors including, Jun proto-oncogene subunit (JunD), reactive species scavengers such as superoxide dismutase 1 (SOD1), and aldehyde dehydrogenase 2 (ALDH2), [18]. These alterations result in eNOS uncoupling which lead to superoxide anion (O2^•−)^ production. The reaction between O2^•−^ and NO produces peroxynitrite (ONOO^−^) [19]. The oxidation of tetrahydrobiopterin (BH4) to dihydrobiopterin (BH2) by ONOO^−^ and hydrogen peroxide (H2O2) further limits eNOS substrate availability and NO production [20]. The uncoupling of eNOS worsens oxidative stress and endothelial dysfunction in a vicious circle. In addition to eNOS uncoupling, the AGEs-induced pathways stimulate an inflammatory cascade response which promotes the expression of growth factors, leading to capillary EC apoptosis, and increased EC permeability. These alterations result in vascular leakage, cytokine response, and leukocyte adhesion which accelerate atherosclerotic plaque progression [21,22].

In diabetic and dysmetabolic patients red blood cells (RBC) alterations have also emerged as mediators of vascular homeostasis dysregulation and endothelial dysfunction. Plasmatic LDL concentrations have been shown to influence the function and structure of RBC, causing a decrease in deformability and increased stiffness [23]. Moreover, altered vascular purinergic signaling has been demonstrated to be involved in the endothelial dysfunction induced by dysfunctional RBC [24]. In addition, in carotid artery plaques, a decreased expression of miR-210 has been demonstrated in RBC leading to increased oxidative stress [25]. Finally, a peroxynitrite-mediated increased arginase activity in RBC has been shown to contribute to endothelial dysfunction [26].

### 2.2. Metabolic Alterations, Oxidative Stress, and Reduced Repair Capacity in Endothelial Dysfunction

Metabolic syndrome is characterized by mitochondrial dysfunction which leads to ROS overproduction due to the impaired function of complex I NADPH (Nicotinamide Adenine Dinucleotide) CoQ reductase and complex III (bc1 complex), to the activation of hypoxia-inducible factor/hypoxia-response element pathway and dipeptidyl peptidase-4 activity as well as to the overwhelming of non-enzymatic defense systems [27]. ROS increase contributes to the reduction in NO bioavailability by enhancing eNOS uncoupling, and impairing endothelial progenitor cells (EPCs) migration, proliferation, and differentiation. The decreased NO production is also associated with impaired synthesis of VEGF which promotes EPCs differentiation, angiogenesis, and vascular repair [28]. Different studies have shown a reduction in EPCs in patients with diabetes or metabolic syndrome [29]. EPCs are circulating components of the endothelium which migrate from the bone marrow and differentiate into mature cells after the stimulation of growth factors to maintain endothelial function and structure after damage [30]. A reduced number of EPCs has been described in diabetic patients [31]. These cells are also characterized by impaired proliferation, migration, and NO-stimulating functions [31]. The number of EPCs is inversely associated with diabetes-related complications, such as micro-angiopathy and macro-angiopathy and to increased brachial-ankle pulse wave velocity [32,33]. Circulating EPCs are also decreased in hypertensive patients with organ damage, contributing to arterial stiffness and increased pulse wave velocity [34].

In subjects with hypercholesterolemia, the exposure of endothelium to oxidized LDL impairs the capacity of EPCs to migrate, adhere, and induce tube formation [35]. In animal models of obesity, the colony-forming capacity of EPCs and their angiogenic capacity in response to ischemic stimulation were significantly altered compared to controls. In contrast, HDL improves EPCs function [36]. The above-mentioned conditions are also associated with a dysregulated ROS production with increased oxidative stress and impaired NO synthesis and availability. Moreover, oxidative stress contributes to increase the secretion of vasoconstrictive factors, such as endothelin-1, thromboxane A2, angiotensin II, and prostaglandin H2, and the production of asymmetric dimethylarginine (ADMA), an endogenous competitive inhibitor of eNOS [37]. On the other hand, antioxidant enzymes including superoxide dismutase, catalase and glutathione peroxidase are reduced in parallel with EPCs [38]. These alterations contribute by increasing blood pressure.

Metabolic syndrome is often characterized by an elevated inflammatory burden which also impacts on EPCs circulating levels and function. In murine models, the knock-out of type-1 interferon receptor and IL-18 has been associated with a restoration of EPCs number with improvement of neoangiogenesis and cell differentiation, whereas increased levels of tumor necrosis factor (TNF) have detrimental effects on EPCs [39,40].

In humans, during metabolic alterations, senescent endothelial cells are increased particularly in aortic arch and coronary arteries and are characterized by reduced NO formation. Moreover, in diabetic and hypertensive patients, the pericapillary basement membrane is significantly thicker, and the proportion of capillaries with disrupted basement membranes between pericytes and EC is higher compared to control subjects [41].

When the endothelium is exposed to detrimental metabolic stimuli including hyperglycemia, hyperlipidemia, and neurohormonal imbalance with increased levels of prostanoids, endothelin and angiotensin-II pathways are activated, leading to endothelial dysfunction and a loss of cell integrity [42]. This cellular stress response has been associated with endothelial-cadherin suppression and with the activation of b1-integrin, p38 mitogen-activated protein kinase (MAPK) and c-Jun N-terminal protein kinase (JNK) signaling pathways that lead to cell apoptosis and arterial denudation [43]. These alterations stimulate smooth muscle cell proliferation, cell growth and migration, matrix secretion and vascular remodeling through the activation of the Ras/Raf/Mitogen-activated protein kinase/ERK kinase/extracellular-signal-regulated kinase (Ras/Raf/MEK/ERK) pathway [43]. ERK impairs insulin receptor substrate-1 and inhibits the phosphatidylinositol 3-kinase (PI3K)/AKT/eNOS pathway [44], further reducing NO bioavailability.

The decrease in NO bioavailability and the increased ROS and peroxynitrite production, as well as the increased levels of thromboxane A2/prostanoids, contribute to the progression of atherosclerotic plaques [43] (Figure 1).

### 2.3. Metabolic Alterations, Endothelial Dysfunction, and Cardiovascular Remodeling

Insulin resistance and hyperglycemia causes an overactivation of the renin–angiotensin–aldosterone system (RAAS) which further contributes to smooth muscle cells proliferation, vascular and cardiac fibrosis, and remodeling, leading to arterial stiffness [45]. Accordingly, patients with metabolic syndrome present increased pulse wave velocity because of arterial stiffness [46]. On the other hand, under hypoxic or inflammatory conditions, endothelial cells enhance the glycolytic flux with the aim being to restore the delivery of oxygen and nutrients to damaged areas [47]. This causes an increased intracellular glucose concentration which promotes the activation of the diacylglycerol/ protein kinase C (DAG/PKC) signaling pathway, enhancing the permeability of the endothelial cell layer and leukocyte adhesion in several tissues, including the heart, retina, and kidney [47]. In these conditions, the secretion of prostaglandins including prostaglandin H2 and F2α (PGH2 and PGF2α) and thromboxane A2 (TXA2) upregulate NAPDH oxidase activity and type 4 and type 5 phosphodiesterases (PDE4 and PDE5) activity, resulting in increased ROS production and cyclic adenosine monophosphate (cAMP) and cyclic guanosine monophosphate (cGMP) degradation [48]. This leads to an impaired response of endothelial-dependent hyperpolarization and increased intracellular free calcium levels, which contributes to vasoconstriction [31,48].

Endothelial dysfunction is also involved in the development of heart failure with preserved ejection (HFpEF) fraction. Interestingly, diabetes, dyslipidemia, hypertension, and metabolic syndrome represent frequent risk factors and comorbidities for HFpEF [49]. In such a context, the reduction in mechano-energetic efficiency index (MEEi), which is the ratio between the left ventricular (LV) work and the whole energy consumption (which corresponds to myocardial oxygen consumption), may precede the development of HFpEF [50]. The association between myocardial MEEi and endothelium*-*dependent vasodilation has been recently demonstrated [51]. Obesity, glucose homeostasis, oxidative stress, inflammation, and insulin resistance may play a role in the development and progression of both endothelial dysfunction and MEEi impairment [51].

Thus, in HFpEF, endothelial dysfunction has emerged as a hallmark of dysmetabolic vascular impairment and contributes to detrimental effects on cardiac metabolism and diastolic dysfunction [52]. In such a context, adiponectin levels in patients with HFpEF are inversely related to flow-mediated dilation, which is a marker of endothelial dysfunction [53]. Furthermore, irisin (a thermogenic hormone that leads to causes energy expenditure by increasing brown adipose tissue) is increased in response to volume and pressure overload, and it has been shown to alleviate cardiac inflammation, oxidative stress, lipotoxicity, apoptosis, myocardial inflammation, and hypertrophic remodeling of the heart [53].

## 3. Biomarkers of Endothelial Dysfunction in Dysmetabolic Conditions

Different biomarkers of endothelial dysfunction have been studied to predict cardiovascular diseases, to identify microvascular and macrovascular damage and the development of atherosclerosis in dysmetabolic conditions. Among them, emerging biomarkers have attracted particular attention with clinical significance and have been proposed for cardiovascular prevention in dysmetabolic conditions (Table 1).

Different cytokines may contribute to the atherosclerotic process and are seen as biomarkers of endothelial dysfunction in dysmetabolic conditions. In the context of atherosclerosis development, endothelial adhesion molecules including E-selectin, P-selectin, intercellular adhesion molecule-1 (ICAM-1), and vascular cell adhesion molecule-1 (VCAM-1) are released in response of proinflammatory cytokines such as interleukin (IL)-1β, IL-6, TNF-α and C-reactive protein (CRP) inducing trans-endothelial migration of leukocytes [54,55,56,57]. In a cohort of patients with atherosclerosis of carotid artery and coronary artery disease, higher levels of E-selectin and ICAM-1 have been found compared to controls [54]. Another study has shown that ICAM-1, IL-6, and CRP may predict lower-extremity atherosclerotic progression [56].

In addition, endothelial microparticles, which are plasma membrane vesicles or plasma membrane blebbing detached from vascular endothelium, are released in the circulation under conditions of endothelial activation such as diabetes, uncontrolled hypertension and hypercholesterolemia that may result in the loss of integrity of endothelium [58]. IL-6 and CRP correlate with circulating microparticles in patients with coronary artery disease [59]. Moreover, endothelial microparticles are significantly higher in patients with myocardial infarction and unstable angina compared to healthy individuals and those with stable angina, suggesting that microparticles may contribute to the development of thrombosis and enhance vascular inflammation [60]. Consistently, endothelial microparticles have been found to be elevated also in peripheral vascular diseases including Takayasu arteritis, microscopic polyangiitis, and polyarteritis nodosa [37]. Endothelial vesicles in the circulation express different CDs including CD62E, CXD31, CD51, CD105, phosphatidylserine, and tissue factor which exert procoagulant, proinflammatory, and proapoptotic functions [61].

Extracellular nano-sized vesicles (EVs), which are released into the circulation system by endothelial cells and platelets after apoptosis, have also been proposed as markers of endothelial dysfunction in diabetic patients with microvascular complications and kidney disease [62].

Other potential biomarkers are represented by oxidized LDL (Ox-LDL), ADMA, and endothelial cell specific molecule-1 (endocan). Ox-LDL activates the CD40/CD40L signaling pathway thus triggering inflammation [62]. In hypertensive patients increased ADMA is associated with increased VCAM-1 and has been suggested as an additional risk factor for coronary artery disease, stroke, and kidney disease. On the other hand, percutaneous coronary intervention has been demonstrated to reduce ADMA levels [63]. Consistently, endocan levels are reduced by adequate risk factor control and correlate with lower levels of ICAM-1 [64].

Other studies have demonstrated that in obese subjects with metabolic syndrome and hypertension endothelial dysfunction may be predicted by the ratio between matrix metalloproteinase 9 (MMP-9) and tissue inhibitor of metalloproteinases (TIMPs) [65]. MMP-7 and MMP-9 induce inflammatory cell migration and invasion into the arterial wall, and contribute to neovascularization, calcification, and the development and destabilization of atherosclerotic plaques [65].

In addition to the abovementioned biomarkers, angiopoietin-like protein-2 (ANGPTL2) is expressed at the level of atherosclerotic plaques promoting monocyte/macrophage chemotaxis and vascular inflammation via the nuclear factor-kappa B (NF-κB) signaling pathway [66]. Consistently, the incidence of cardiovascular events has been shown to correlate with ANGPTL2 concentrations also after adjustment for conventional risk factors [67].

In patients with dyslipidemia and hypertension, the expression of endoglin is increased, and it is associated with endothelial dysfunction, adhesion, and migration of inflammatory cells and development of atherosclerotic lesions. Endoglin is a type-I transmembrane glycoprotein and functions as a coreceptor for ligands of the transforming growth factor (TGF)-β superfamily [68]. Endoglin is predominantly expressed by activated endothelial cells and plays a crucial role in angiogenesis. Treatment with statins has been shown to reduce endoglin levels in parallel with cholesterol and plaque size [68].

In an animal model of obesity, an increased expression of senescence markers (including p53, p21, p16), tissue factor, VCAM-1, and sodium glucose transporter (SGLT) 1 and 2 has been detected in the aortic inner curvature, corresponding to a reduced expression of eNOS [69].

**Table 1:** Biomarkers of endothelial dysfunction.

Biomarkers of Endothelial Dysfunction	Characteristics
E-selectin and ICAM-1 [54]	High levels in carotid artery and coronary artery disease
ICAM-1, IL-6, and CRP [56]	Prediction of lower-extremity atherosclerotic progression
Endothelial microparticles [59]	Elevated levels in myocardial infarction, unstable angina, peripheral vascular diseases including Takayasu arteritis, microscopic polyangiitis, and polyarteritis nodosa
Endothelial vesicles expressing CD62E, CXD31, CD51, CD105, phosphatidylserine, and tissue factor [61]	Procoagulant, proinflammatory, and proapoptotic functions
ADMA [63]	Additional risk factor for coronary artery disease, stroke, and kidney disease
MMP-7 and MMP-9 [65]	Inflammatory cell migration and invasion into the arterial wall, neovascularization, development, and destabilization of atherosclerotic plaques
ANGPTL2 [66,67]	Monocyte/macrophage chemotaxis and vascular inflammation at the level of atherosclerotic plaques Correlation with cardiovascular events
Endoglin [68]	Adhesion and migration of inflammatory cells and development of atherosclerotic lesions

## 4. Cardiometabolic Drugs and Endothelial Function

Novel antidiabetic drugs may improve endothelial function. In particular, treatment with the SGLT2 inhibitor empagliflozin reduced body weight, perirenal fat, and glucose levels by 28%, and improved endothelial function, cardiac remodeling, cardiotoxic lipids, and autophagy [69]. Moreover, empagliflozin decreases aortic stiffness, renal resistivity index, and kidney injury [69]. Empagliflozin treatment reduced the expression of p53, p21, and p16 in the inner curvature of the aortic arch at comparable levels to those in lean animals [69]. In the mesenteric artery empagliflozin restored NO-mediated endothelial relaxation and blunted endothelium-dependent contractile responses to acetylcholine preventing the formation of cyclooxygenase-derived contractile prostanoids. Empagliflozin normalized heart weight, improved left ventricle weight and volume, and reduced the posterior wall thickness [69]. In another study canagliflozin has been demonstrated to increase relaxation in coronary arteries from diabetic mice as a consequence of vascular smooth muscle cells inhibition [70].

Glucagon like peptide-1 receptor agonists (GLP1-RA) have also been demonstrated to exert protective effects on the endothelium, vascular tone, and inflammation. GLP1-RA may contribute to the stimulation of eNOS through the activation of the protein kinase AMP-activated catalytic subunit alpha (AMPK)/Akt pathway [71]. This class of drugs increase the expression of sirtuins and reduce the adhesion of monocytes. Moreover, they suppress the expression of proinflammatory cytokines and chemokines, and they regulate the adiponectin/APPL1 signaling pathway. All these actions may contribute to preventing atherosclerosis progression and plaque vulnerability in dysmetabolic patients [71].

Drugs commonly used to reduce high cholesterol levels in dysmetabolic patients, such as statins, have also been largely demonstrated to exert vascular protective effects restoring endothelial NO production, increasing the expression of tissue-type plasminogen activator (t-PA) and attenuating P-selectin expression and leukocyte adherence [72]. Moreover, PCSK9 monoclonal antibodies have been shown to improve endothelial function, since PCSK9 inhibitors may attenuate the proinflammatory activation of endothelial cells, increase circulating EPCs and promote vascular repair independently from LDL cholesterol regulation [73].

## 5. Endothelial Dysfunction in COVID-19 with Associated Metabolic Comorbidities

**Figure 2:**
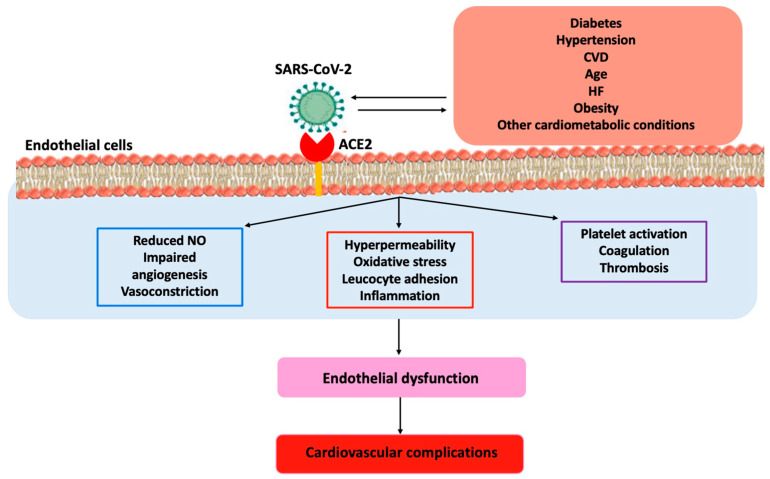
Endothelial dysfunction during SARS-CoV-2 infection. Figure legend: ACE2, angiotensin converting enzyme 2; CVD, cardiovascular diseases; HF, heart failure; NO, nitric oxide.

There is a bidirectional pathophysiologic relationship between Coronavirus Disease 2019 (COVID-19) and cardiometabolic diseases [74,75]. A large body of evidence has shown that patients with cardiometabolic conditions, in particular obesity, hypertension, diabetes mellitus, and heart failure, present an increased risk for severe forms of COVID-19 associated with a high rate of death [74,75]. It has been estimated that nearly 63.5% of hospitalizations for severe COVID-19 were attributable to the coexistence of cardiometabolic conditions [76]. On the other hand, patients with severe COVID-19 infection are more prone to develop cardiovascular complications [74,75]. In such a context, endothelial dysfunction plays an important role in the development of severe COVID-19 disease after SARS-CoV-2 infection. Indeed, although SARS-CoV-2 infection primary involves the respiratory system, endothelial damage has been described as a hallmark of cardiovascular complications, particularly in dysmetabolic patients [77]. The damage of endothelium in COVID-19 consists of endotheliopathy and endotheliitis, causing a proinflammatory and procoagulant state [77] (Figure 2).

The virus S-protein binds ACE2 and activates the type 1 angiotensin II receptor (AT1R). Moreover, the nucleocapsid binds the NOD-like receptor protein 3 (NLRP3) inflammasome in host cells leading to the formation of pores in cell membranes and to the release of cytosolic content, which results in cell death [78]. The trigger of NLRP3 inflammasome contributes to induce and perpetuate the cytokine storm syndrome (CSS), consisting of hyperinflammation, due to the overproduction of cytokines, responsible for systemic damage in the lung, brain, heart, and kidney [79]. CCS is associated with endothelial damage in the microvasculature and to a systemic dysregulation of the balance between fibrinolysis and coagulation [79]. The stimulation of AT1R causes an overactivation of NADPH-oxidase (Nox) 2, one of the most important cellular producers of ROS [80].

During severe COVID-19, the carbohydrate-rich layer lining the endothelium (the endothelial glycocalyx) is damaged along with a consequent capillary permeability, leukocyte adhesion, and a procoagulant state [81].

Moreover, a consequence of the endothelium activation is represented by the release of endothelial cells and vesicles into the bloodstream. EVs expressing CD62 (E-selectin) have been shown to be related to the severity of the disease at admission and to in-hospital mortality [82].

Consistently, patients presenting conditions associated with high risk of having endothelial dysfunction (such as aging, smoke, hypertension, diabetes, previous cardiovascular events, heart failure) present a higher risk of severe COVID-19 disease [83,84,85,86].

On the other hand, acute systemic infections may destabilize chronic cardiovascular conditions contributing to coronary plaque rupture, thrombosis, and heart failure [87].

The increased production of ROS, the reduction in NO and the enhanced activity of angiotensin II have also been suggested to play a role in the development of chronic hypoxia and long COVID [88].

Chronic hypoxia indeed produces vascular changes, pulmonary fibrosis and hypertension, arterial stiffness, and decreased lung function [89]. Several weeks after SARS-CoV-2 infection, patients who develop long COVID have reduced reactive hyperemia index (RHI) and reduced flow-mediated dilation that may represent a surrogate marker of persistent endothelial inflammation [90].

Chronic endothelial inflammation is associated with increased levels of procoagulant factors including factor VIII, plasminogen activator inhibitor-1 (PAI-1), von Willebrand factor (vWF), thrombomodulin, ICAM-1, VCAM-1, P-selectin, and D-dimer [91]. Moreover, in COVID-19 patients, an acquired natural anticoagulant deficiency has been described. This condition consists of the decrease in protein C activity and free protein S and in a higher prevalence anticardiolipin IgM antibodies levels [91].

An overwhelming presence of procoagulant factors has been detected in the vascular endothelium from autopsies of COVID-19 patients, suggesting a role of activation of endothelial cells and platelets [92]. Moreover, the systemic infection has been shown to reduce the transcription of thrombomodulin, a transmembrane glycoprotein expressed on endothelial cells, which physiologically deactivates thrombin and the anticoagulation cascade [93].

Another described mechanism consisting in the dysregulation of the kallikrein-kinin system (KKS), which normally counterbalances the renin–angiotensin–aldosterone system, contributes to vasodilation, regulates inflammation and coagulation through the release of bradykinin and other vasoactive peptides [94].

In such a context, a higher prevalence of acute coronary syndromes, acute pulmonary embolism, deep veinous thrombosis and ischemic stroke has been reported as a consequence of the persistence of inflammation, cytokine storm, microvascular damage, and stress-induced cardiomyopathy [95].

These findings are more frequent in patients with dysmetabolic conditions, advanced age (>75 years), with chronic kidney disease and history of coronary, carotid, peripheral arterial disease, or venous thromboembolism [96].

Chronic cardiac inflammation may lead to the development of fibrosis, increased stiffness, reduced myocardial perfusion, and arrhythmias [97].

## 6. Conclusions

Cardiometabolic disorders are associated with structural and functional alterations of the endothelium and with an increased risk of major cardiovascular events. Endothelial dysfunction also plays an important role in the development of severe COVID-19 disease and cardiovascular complications in dysmetabolic patients after severe SARS-CoV-2 infection. In the context of metabolic disorders, different biomarkers of endothelial dysfunction have been proposed to identify microvascular and macrovascular damage, to early detect atherosclerotic progression, and possibly to predict cardiovascular diseases.

Among new antidiabetic drugs, SGLT2i and GLP1-RA have been demonstrated to improve endothelial function, cardiac remodeling, cardiotoxic lipids and autophagy and may represent a first-line therapeutic strategy in the management of cardiometabolic disorders.
